# The Substance That Forms the Skeleton and Teeth—or, Development of the Teeth

**Published:** 1882-12

**Authors:** G. W. Smith


### The Substance that Forms the Skeleton and Teeth—or, Development of the Teeth.

By Dr. G. W. Smith.

[Read before the Cincinnati Dental Society, October 10, 1882.]

The principle on which the skeleton is formed from the lowest grade of animal to the highest type consists of a fluid with granuels and cells. In the course of development tubular tracts are formed, some of which are filled with neurine, or nervous matter, others with myoline, or muscular matter, other portions are converted into glanlular substance, while a great portion of the primordial substance goes to forming cellular matter. This substance, according to Owen, in many animals becames hardened in certain parts of the body by earthy salts, and when these salts consist chiefly of phosphate of lime, this tissue is called osteone or bone, and dentine or tooth, and is established between which the chief separation may be found in the manner of the earthy particles, relative to the maintenance to a free circulation more or less of nutrition through such dense, or hardened tissue. We find in bone certain openings like canals and their size are sufficient to admit capillary blood-vessels through this calcified tissue. And again, we have minute tubes, many of which expand into cell-like cavities, these tubes and cells are established for the slower circulation or percolation of the colorless fluid of the blood, plasma, or liquor sanguinis. In dentine of a complete character there is a provision made, tubes of minute dimensions are established for the passage of plasma through the tissue, but the particles of red blood through these openings are excluded. Mature or true bone and dentine belong to the highest order, or "division of the animal kingdom."

This province has received the term vertebrata, from the predominating, or prevalent character of the bone matter, in successive groups of more or less confluent bones called vertebræ. Before leaving this part of the subject to take up the development of the teeth it will, perhaps, be well to mention the composition of that earthy constituent in making up the different classes of vertebrate animals. We will, for example, mention four classes, namely, fish, birds, reptiles and mammals, the last, however, belong to the hair-clad beasts of the quadruped family. These four classes as well as others that belong to the animal kingdom, from the lowest order to the highest type, are but the production of organic and inorganic matter, and the earthy constituent varies according to the class represented.

In the fish tribe will be found the smallest portion of the earthy matter in their bones, birds, however, have the largest percentage of these classes of which I have mentioned, but the organic or animal part in all will consist chiefly of a "gelatinous substance." Out of this earthy constituent, which is the original substance, we have the formation of bone and tooth, and this is the acknowledged principle upon which nature produces the frame-work of all osseous development.

I will now confine myself more closely to the formation of the human teeth:

The anatomical division of a tooth may be divided into three parts. The crown, the cervix, the root, the latter is the portion imbedded in the alveolus. This organ embraces four distinct parts. The first is the soft vascular part, termed pulp, which is situated in the center of the dentine, the second part is bone, or dentine, which forms the larger portion of the tooth, the third tissue, which is the enamel, forms the covering or protection to the organ. The last of these four parts is the cementum, which covers the root of the tooth and is thickest at the apex. The chemical composition of teeth may be found as follows:

Dentine, 66-72 per cent.

Enamel, 89-82 per cent.

Phosphate of lime, with a truce of fluate of lime.

Carbonate of lime, 3-36 per cent.

Phosphate of magnesia, 1-08 per cent.

Salts " " 00.83 per cent.

Chondrine " 27-61 per cent.

Fat, 00-40 per cent.

This part of the osseous development is the crowning effort in the production of bone tissue, its deposits while in their granular form are of the finest earthy constituents and its texture is not only the hardest of all animal development but the most beautiful in construction of any of the hard tissues of the body. The first state of development in a tooth is the papellæ, which may represent a small cone-like eminence situated at the bottom of the dental grove, which forms the germs of the dental organs in their first or primitive stage of development. The blood globules floating in their plasmatic condition are just beginning to see the dawn of their nucleated state or the organization of the elementary cells, out of which the vascular pulp is formed.

The capsule through process of development will leave the summit and descend into the spaces between the dentinal pulp plates, and will also resemble them in form, and they are found to adhere to the so-called base of the surface of the capsule next the mouth, and also by their lateral margins to the sides of the capsule, which resembles walls—like partitions separating each dental plate and confining it to its proper chamber; but the margin of the partition opposite its attached base is free in the interspaces of the origin of the dental pulp plate. The tooth in a gelatinous or pulpy formation is full size in each particular part of development before calcification takes place. Yet the tooth may not be perfect in dimensions, but it is a fact beyond doubt that each part assumes a pulpy condition even to the cusps and cutting edges before calcification takes place. This hardening of the earthy salts commences at the outer surface of the lamelliform process, which is farthest from the attached base. Upon each of the processes there is found a little cap at the commencement, into which the pulp plate edges are separated. As the centripetal calcification and conversion takes place, these all when farther developed unite in one single transverse plate, and the process of conversion having reached the base of the pulp plates, these plates then coalesce and form the base to the crown of the organ.

The enamel organ which Cuvier appears to have noticed, under the name of the internal layer of the capsules, may be distinguished by its color, which is of a light blue, sub-transparent texture, adhering to the free surface of the partition, formed by the true inner layer of the capsule.

Before the commencement of the formation of the teeth, the enamel pulp is found in close contact with the dentine pulp. Yet there is a distinct separation or vacuity between them which follows uninterruptedly between all the gelatinous plates in connection with the dentine pulp, and also by the division formed by the uniting of the enamel pulp and the folding of the capsule.

According to the excretion theory this very delicate arrangement must have been subjected at once to the violence of the compress or unyielding or bony box, by which the deposition of the hard substance of the organ in the conditional, or hypothetical vacuity, between the enamel crust and dental pulp.

Cuvier says a process of absorption must have been conceived to be set on foot immediately, that the altered condition of the gelatinous secreting organs took place, and, according to the hypothesis, the secreting function must be supposed to have proceeded without irregularity or interruption, while the process of absorption was superinduced in the same part to relieve it from the effect of pressure produced by its own secretion.

The dentine commences its formation immediately under the enamel and can readily be traced by the grayish ring or line that divides the enamel from the dentine. This bone tissue is not so dense as the enamel but its constituent is a well organized foundation disposed in form of minute tubes and cells, filled with earthy constituent which have a two-fold disposition of its arrangement in either blending with the organic substance of the tubes and cells, or it may be held in a minute granular form in their cavities.

The texture of the bone tissue that lies next the enamel, which is dentine, arises mostly from the proportions of earthy salts in the first or primitive state of combination. The tubes and dental cells hold within their walls beside the earthy matter the fluid portion of the blood called plasm, or liquor-sanguinis, which is relative to the mechanical conditions of the tooth, and to the vitality and nourishing of the dentine. In matured dentine the tubes diverge from the center of the tooth leaving the pulp cavity they pass to the outer surface of the organ, their course being wavy, at right angles from the hollow of the tooth.

The hardened tissues of the teeth are disposed in hollow columns, they are erect to the plain of pressure and they possess a certain elasticity which results from their curves, they are perpendicular where the appulse of the crown receives the opposing tooth and are parellel where they come in contact with the contiguous teeth. If a small section of dentine be submitted to a highly magnified power it will show the tubuli with the inter-tubular substance in its primary condition, and the mode in which the nucleated cells are changed into the basis of the tooth or tooth pulp.

As has been before stated the tubes, or tubuli, beside fulfilling the mechanical end, above mentioned, receive the infiltration of the plasm as it passes from the remains of the vascular pulp and is transuded through the dentine by the anastomosing branches of the tubuli, and by the plasmatic cells of the inter-tubular substance, through which the tissue maintains sufficient circulation to vitalize the organs. On the pulp surface we have minute nerve fibers radiating outward from within causing sensations of acuteness, which most every one has felt when decay has reached the dentine or when mechanical or chemical stimuli sets the tooth on edge ; but matured dentine has no canals of sufficient size to admit capillary blood-vessels containing the red particles of blood. The first modified condition of dentine is where the capillary constitutent of the vascular pulp in its elementary or primitive condition remains uncalcified and is the medium through which the red blood finds its way into all the substances of the tissue. The next modification is found in the fundamental tissue of the dentine where the cellular basis is disposed in concentric layers about the vessels and canals, which contain radiated cells like those present in the osseous substance, which is called osteo-dentine.

"The transition from dentine to vaso-dentine and from this to osteo-dentine is gradual and the resemblance of osteo-dentine to true bone is very close." So that composed teeth are the results of certain earthy salts, and earthy salts, in chemical language, is but the example of a class of inorganic elements. "Salts, as denominated in chemistry, is a combination which does not consist of two elements, but a combination of two parts each containing two elements."

